# Die palpablen Gebilde des normalen menschlichen Körpers und deren Methodische Palpation

**Published:** 1906-09

**Authors:** 


**Die palpablen Gebilde des normalen menschlichen Körpers und deren Methodische Palpation.** Von Dr. Toby Cohn. 1. Teil: Obere Extremität. Pp. viii., 216. Berlin: S. Karger. 1905.

The writer of this volume is convinced that, compared with what it might be, palpation of the human body is an undeveloped art, though an art which when acquired is of extreme value for diagnostic and therapeutical purposes.

The present volume, dealing with the upper extremity, is the first of a series he is writing in which palpation is discussed in a most complete manner. We admit that in no other work have we seen such a complete account of the anatomy of the upper extremity as revealed to the sight and touch of the skilled palpator.

Works on artistic anatomy by no means cover the same ground as this work, for they are concerned only with the structures which are made manifest by projection through the skin, and appeal solely to the sense of sight and leave unconsidered a great deal which is easily revealed by palpation.

We have nothing but praise for the thorough manner in which the author places before the reader the principles, technique, and practical application of palpation to the upper extremity, and the twenty-one original illustrations add considerably to the value of the work. We venture to think that it will be a standard book of reference for those who wish to thoroughly study "superficial anatomy" and massage.

